# Irreversible glacier change and trough water for centuries after overshooting 1.5 °C

**DOI:** 10.1038/s41558-025-02318-w

**Published:** 2025-05-19

**Authors:** Lilian Schuster, Fabien Maussion, David R. Rounce, Lizz Ultee, Patrick Schmitt, Fabrice Lacroix, Thomas L. Frölicher, Carl-Friedrich Schleussner

**Affiliations:** 1 Department of Atmospheric and Cryospheric Sciences (ACINN), Univ. Innsbruck, Innsbruck, Austria; 2 Bristol Glaciology Centre, School of Geographical Sciences, Univ. of Bristol, Bristol, UK; 3 Department of Civil and Environmental Engineering, Carnegie Mellon Univ., Pittsburgh, PA USA; 4 Cryospheric Science Lab, NASA Goddard Space Flight Center, Greenbelt, MD USA; 5 GESTAR-II Cooperative Agreement, Morgan State Univ., Baltimore, MD USA; 6 Climate and Environmental Physics, Univ. of Bern, Bern, Switzerland; 7 Oeschger Centre for Climate Change Research, Univ. of Bern, Bern, Switzerland; 8 Institute of Geography, Univ. of Bern, Bern, Switzerland; 9 Energy, Climate and Environment Program, International Institute for Applied Systems Analysis (IIASA), Laxenburg, Austria; 10 Geography Department and Integrative Research Institute on Transformations of Human-Environment Systems (IRI THESys), Humboldt Univ. of Berlin, Berlin, Germany

**Keywords:** Hydrology, Cryospheric science, Climate-change impacts, Projection and prediction, Water resources

## Abstract

Exceeding 1.5 °C of global warming above pre-industrial levels has become a distinct possibility, yet the consequences of such an overshoot for mountain glaciers and their contribution to raising sea levels and impacting water availability are not well understood. Here we show that exceeding and then returning to below 1.5 °C will have irreversible consequences for glacier mass and runoff over centuries. Global climate and glacier simulations project that a 3.0 °C peak-and-decline scenario will lead to 11% more global glacier mass loss by 2500 compared with limiting warming to 1.5 °C without overshooting. In basins where glaciers regrow after peak temperature, glacier runoff reduces further than if the glaciers stabilize, a phenomenon we call ‘trough water’. Half the studied glaciated basins show reduced glacier runoff with overshoot compared with without for decades to centuries after peak warming. These findings underscore the urgency of near-term emissions reductions and limiting temperature overshoot.

## Abstract (web summary)

How mountain glaciers will react to temporarily overshooting 1.5 °C of warming is poorly understood. Here the authors show irreversible global glacier loss for centuries after overshoot, implying long-term reductions in glacial water resources with amplified impacts in regions where glaciers regrow.

## Main

The loss of mountain glaciers contributes to sea-level rise^1^, influences water resources in periods of drought or low precipitation^2^ and induces natural hazards^3,4^. Glaciers are projected to continue losing mass through the twenty-first century^5^ and beyond^6^, with the extent and pace of mass loss mainly driven by the increase in surface temperature. Global temperatures are rising at a record rate^7^ and, as a result of past inaction and insufficient emissions reductions, today, the risk of exceeding a global mean temperature level of +1.5 °C above pre-industrial levels as early as in the next decade is increasing^1,8^. In light of such an overshoot, substantial carbon removals will be required to reverse the warming to back below 1.5 °C over the long term^9^. The consequences of such an overshoot on the climate, ecosystems and societies^10^, as well as on hydrology and water resources^11^, are not well understood. Even if the global mean temperature returns to the target temperature, regional temperatures may not^10,12,13^. The magnitude and duration of the overshoot also matters. For example, the time spent above 1.5 °C is quasi-linearly related to projected sea-level rise in 2300^14^ and will affect future emissions from other systems that respond more slowly (for example, permafrost and peatlands)^12^. Furthermore, global warming overshoots increase the risks of crossing potential tipping point thresholds^15–18^, which may lead to irreversible glacier changes^19^. However, if the local climate and characteristics are favourable, glaciers can regrow. Notable examples include the recent Little Ice Age period in Central Europe^20^ or the present-day regrowth of Crater Glacier, United States, following the eruption of Mount St. Helens^21^. Given that glaciers respond slowly to climate change and that regrowth takes longer than retreat^22^, the impacts of overshoot scenarios are likely to be long-lasting.

One challenge with quantifying overshoot impacts is a lack of suitable climate projections. Only a few climate models that participated in the sixth phase of the Coupled Model Intercomparison Project (CMIP6) have run global temperature overshoot simulations^23^ (Supplementary Fig. 1). Moreover, these simulations end in 2300, which is too early to capture glacier regrowth. Furthermore, these simulations lack corresponding reference temperature stabilization scenarios for comparisons because regional temperatures may evolve even under stabilized global mean temperatures. Climate simulations from the GFDL-ESM2M Earth System Model (ESM)^24,25^ address this gap by using an adaptive emissions reduction approach^26^ to modelling different stabilization and temporary overshoot scenarios for 1.5 °C through 2500^13^.

Here we conducted a systematic analysis of irreversible glacier changes and the associated impacts for all (more than 200,000) mountain glaciers globally under temperature stabilization and overshoot scenarios. Forcing from both idealized experiments and ESM-generated stabilization and overshoot scenarios through 2500 was used in conjunction with the Open Global Glacier Model (OGGM)^27^ to reveal key drivers of change and characteristic time scales that vary regionally. We found that the potential regrowth of glaciers after a temperature overshoot causes a temporary depletion in glacier runoff, which we refer to as ‘trough water’. We highlight the regional variability and the potential future impacts of trough water for select glaciated basins. Overall, our projections reveal that long-term irreversible glacier mass loss is highly sensitive to the magnitude of the local temperature overshoot, and that avoiding overshoot results in a reduced glacier contribution to sea-level rise and smaller changes to water availability.

## Fast- versus slow-responding glacier mass and runoff response

**Fig. 1:**
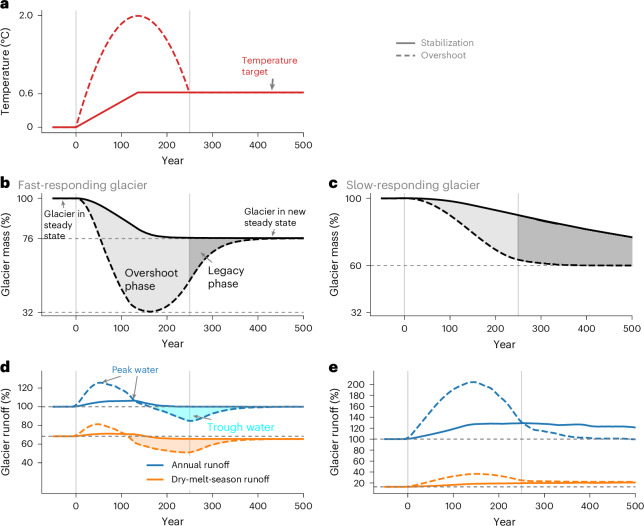
Projected mass and runoff change for a fast- and slow-responding glacier under idealized temperature stabilization and overshoot experiments. **a**–**e**, Applied temperature change (**a**), glacier mass relative to the initial state (**b**,**c**) and glacier runoff relative to the initial state (**d**,**e**) for a typical fast-responding glacier in the mid-latitudes (Aletsch Glacier) (**b**,**d**) and a slower-responding glacier in the high latitudes (Wykeham Glacier South) (**c**,**e**). The overshoot phase refers to when the temperature exceeds the stabilization temperature, and the legacy phase refers to the period of time after the temperature stabilizes until the glacier reaches its new steady state. Precipitation is kept constant. Dry-melt-season runoff refers to the three driest months over the melt season and is shown relative to the annual initial glacier runoff. The runoff *y*-axis scales in **d** and **e** are different. Idealized changes for the largest glaciers in five other regions are shown in Extended Data Fig. 1. See Methods for additional detail on the idealized experiments.

**Extended Data Fig. 1:**
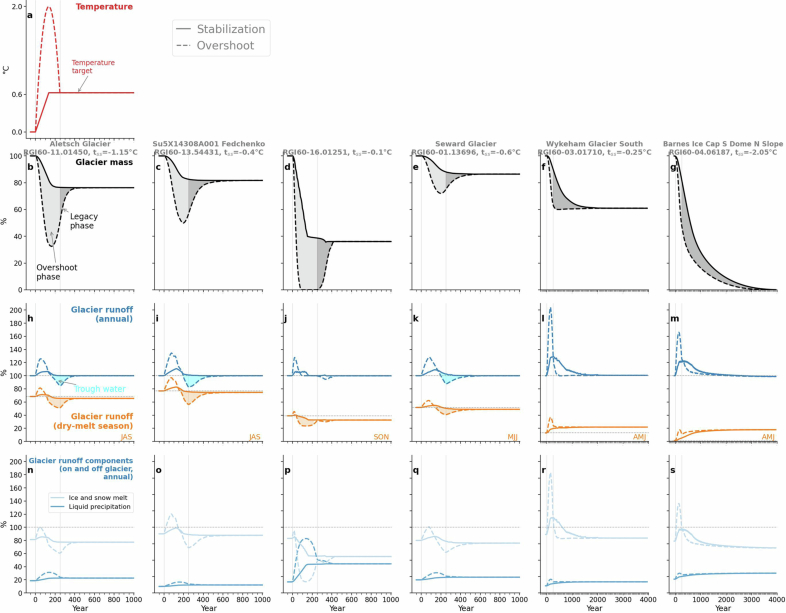
Projected glacier mass and runoff change for idealized temperature stabilization and overshoot experiments (similar to Fig. 1, but for the largest glaciers of six glacier regions). (**a**) Applied temperature change. (**b-g**) Glacier mass remaining, and (**h-m**) annual and seasonal glacier runoff response, both relative to the initial state. (**n-s**) Annual glacier runoff components from the initially glacierised area given as the ice and snow melt and liquid precipitation. The steady-state temperature bias t_ss_ is given for every glacier. Precipitation is kept constant. Dry-melt season runoff refers to the three driest months over the melt season and is shown relative to the annual initial glacier runoff. Annual and seasonal runoff is 21-year averaged. Note that for four glaciers, we show the experiments only for 1000 years, while the very slow-responding Wykeham Glacier South and Barnes Ice Cap, are shown for 4000 years. See Supplementary Information for additional detail on idealized experiments.

Glacier characteristics strongly shape their response under overshoot. We illustrate this by comparing the idealized temperature overshoot and stabilization experiments for two glaciers with different climatic settings and response times—the fast-responding Aletsch Glacier and the slow-responding Wykeham Glacier South. The pace and timing of the temperature increase, as well as the occurrence of an overshoot, result in considerably different glacier mass and runoff projections (Fig. 1). The rate of temperature increase drives the mass loss rate, although the relative changes vary considerably between the fast- and slow-responding glacier (Fig. 1a–c). The temperature overshoot causes both glaciers to lose substantially more mass during the overshoot phase, which the fast-responding glacier takes decades to centuries to recover from (Fig. 1b). The slow-responding glacier does not recover by the end of the idealized 500-year simulation (Fig. 1c), but the experiments converge to the same steady state after millennia (Extended Data Fig. 1).

In steady state, the glacier mass and glacier runoff (comprising the meltwater plus liquid precipitation from the initially glacierised area) are constant (Fig. 1d,e). When the temperature increases, the glaciers begin losing mass, and annual as well as dry-melt-season runoff increases. As the glaciers retreat and the mass loss rates decrease, a maximum annual and dry-melt-season runoff is reached, referred to as ‘peak water’^28^. After decades to centuries, the annual runoff returns to the same level as in the initial state, although the seasonal distribution of runoff may change.

The differences between the experiments over the first century show that the magnitude of the peak water is considerably smaller when the temperature and glacier mass changes are slower. For faster-responding glaciers, the overshoot experiment highlights a unique phenomenon in which annual glacier runoff temporarily decreases below the initial level as the glacier regrows during the temperature decline phase (Fig. 1d). As a response to glacier regrowth, water available from the initially glacierised area is reduced over a certain period—trough water (as opposed to peak water)^28,29^.

The influence of a temperature overshoot on a given glacier depends on its response time (Fig. 1 and Extended Data Fig. 1), which depends on local characteristics, such as local temperature change, the glacier surface slope and/or the mass-balance gradient^22,30^. These model results under idealized climate scenarios suggest glacier change is reversible for a typical mountain glacier if the glacier is given enough time. In reality, precipitation also changes with increasing temperatures (Supplementary Fig. 2). Thus, trough water can occur in the absence of glacier regrowth. Glaciers are also not in a steady state. Hence, ESM simulations for overshoot and corresponding stabilization scenarios are needed to quantify the time needed for them to regrow or converge and the associated consequences for water availability.

## Global glacier mass response

**Fig. 2:**
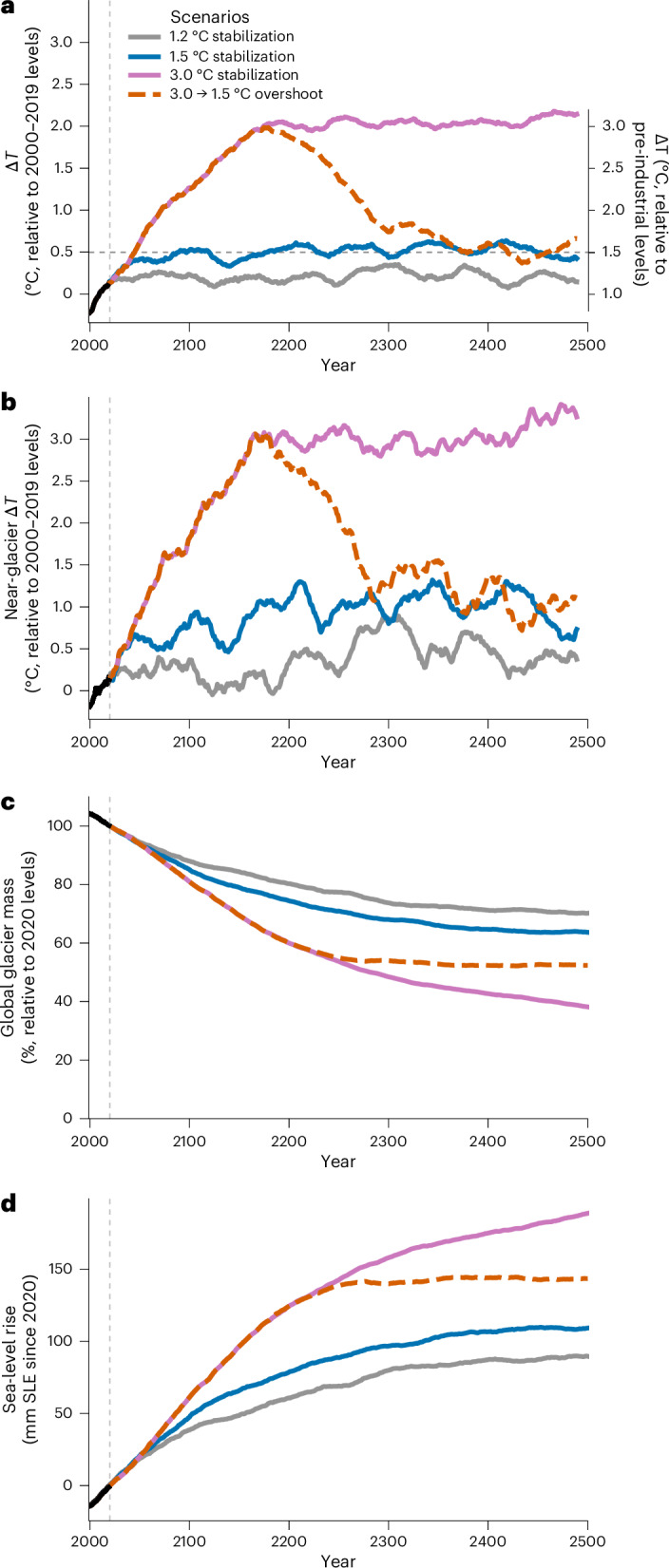
Projected air temperature and glacier mass change for stabilization and overshoot scenarios of the GFDL-ESM2M climate model. **a**, Global mean surface air temperature change *ΔT* relative to the 2000–2019 average (left *y* axis) and above pre-industrial levels, as defined by the IPCC AR6 (ref. ^1^) (right *y* axis). **b**, Global glacier-area weighted near-glacier temperature change relative to 2000–2019. **c**,**d**, Global glacier mass remaining (**c**) and sea-level rise contributed by glaciers (**d**) for the same scenarios. Time series in **a** and **b** are smoothed with a 21-year centred rolling mean. Past changes from 2000–2019 are shown in black. The global precipitation changes are provided in Supplementary Fig. 9.

Stabilization and overshoot scenarios from the GFDL-ESM2M were used to force the OGGM and quantify the mass changes in all glaciers globally from 2000 to 2500 (Fig. 2). Although our applied scenarios resemble our idealized temperature change experiments, the global projections explicitly account for local and regional variations in temperature and precipitation. Specifically, the local projected signal differs from the global temperature overshoot signal because the glacier-covered areas are, on average, subject to greater temperature change than the global mean. For example, the glacier-area weighted temperature is up to 1 °C warmer than the global mean for the 3.0 °C stabilization scenario, consistent with previous findings^1^.

**Extended Data Fig. 2:**
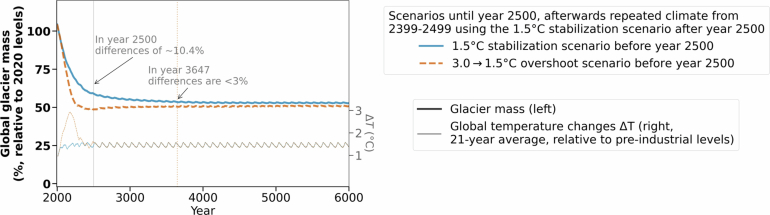
Global glacier mass projections from the year 2000 until 6000 (% relative to 2020) under an extended 1.5^∘^C stabilization and 3.0 → 1.5^∘^C overshoot scenario. Until 2500 the same scenarios as in Fig. 2a were applied. After 2500, an identical climate (randomly repeated climate taken from the last 100 years of the 1.5^∘^C stabilization scenario) was used. The resulting global mean temperature changes of the extended scenarios are shown. For visual reasons, we only show the simulations until year 6000 as no notable changes occur afterwards, that is the global glacier mass reached steady state in both scenarios.

Even if global warming ceased today at 1.2 °C above pre-industrial levels^7^, the world’s glaciers would still lose 30% of their mass by 2500, relative to 2020 (Fig. 2c), corresponding to an additional 90-mm sea-level equivalent (SLE) (Fig. 2d). Under the 3.0 → 1.5 °C overshoot, glaciers temporarily lose up to 16% more mass (50-mm SLE) than with 1.5 °C stabilization. Mass loss occurs more quickly in the overshoot scenario. After 2250, glacier mass loss slows and almost halts in the 3.0 → 1.5 °C overshoot, whereas long-term mass loss continues in the 1.5 °C stabilization. Although the influence of the overshoot diminishes over time, the 3.0 → 1.5 °C overshoot still results in 11% more global glacier mass loss in 2500 (34-mm SLE). The glacier contribution to sea-level rise is never negative, even in the 3.0 → 1.5 °C overshoot scenario. Beyond 2500, glaciers would continue to lose an additional 6% mass under 1.5 °C stabilization (Extended Data Fig. 2). Under the 3.0 → 1.5 °C overshoot scenario, glaciers stabilize faster because enhanced mass loss occurs due to the stronger temporary forcing during overshoot. These results confirm that overshoot-induced global glacier mass loss, and the corresponding contribution to sea-level rise, is irreversible for centuries to millennia post-overshoot.

## Regional glacier mass response

**Fig. 3:**
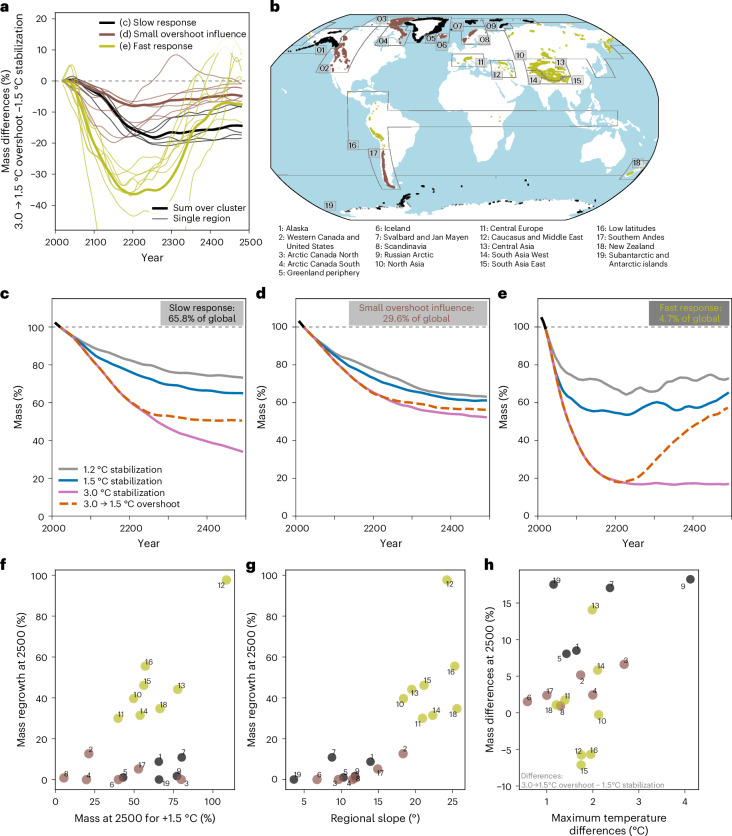
Regional glacier mass response under stabilization and overshoot scenarios of the GFDL-ESM2M climate model. **a**, Regional mass differences between the 3.0 → 1.5 °C overshoot and the 1.5 °C stabilization scenario for all 19 glacier regions and the sum over the three clusters. Clusters were selected from these mass differences and represent regions with similar overshoot influence on mass change (Methods). **b**, Nineteen glacier regions colour-coded by the three clusters, with dots showing glacier locations. **c**–**e**, Glacier mass projections for the sum of each cluster with a slow response (**c**), a small overshoot influence (**d**) and a fast response (**e**). The percent refers to the cluster’s fraction of the 2020 global mass. **f**,**g**, Regrowth after 3.0 → 1.5 °C overshoot described by the year 2500 minus the minimum glacier mass versus the glacier mass in 2500 under 1.5 °C stabilization (**f**) and the regional glacier-area weighted glacier surface slope (**g**). **h**, Mass difference in 2500 between 1.5 °C stabilization and 3.0 → 1.5 °C overshoot versus the maximum regional temperature difference of the same scenarios. Numbers are given as a 21-year centred rolling mean in percent relative to 2020. Past changes from 2000 to 2019 are shown in black in **c**–**e**. All scenarios and regions are shown individually with their 2020 glacier mass in Extended Data Fig. 3. Basemap in **b** from Natural Earth (https://www.naturalearthdata.com/).

**Extended Data Fig. 3:**
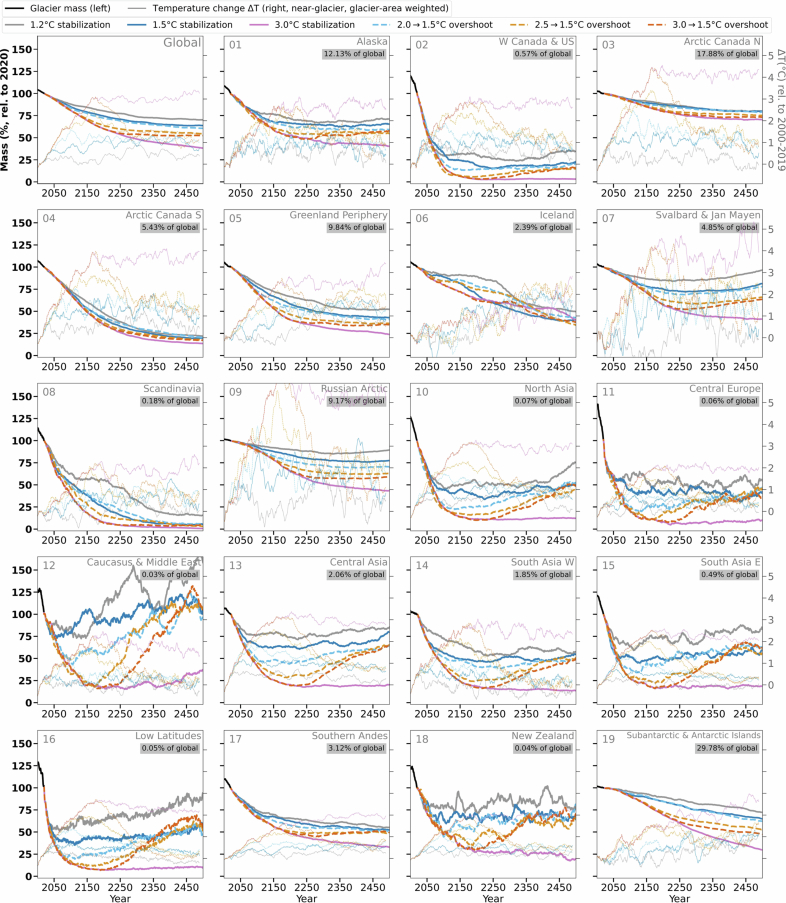
Glacier mass remaining under stabilization and overshoot scenarios together with regional near-glacier temperature changes (21-year averaged) for every RGI region. Glacier mass in 2020 relative to global estimates is given globally and for every region. Past changes from 2000 to 2019 are shown in black. Glacier regions’ locations, their clusters and other characteristics are given in Fig. 3.

Global mass change is dominated by the regions with the most glacier mass, which have slow response times (Fig. 3 and Extended Data Fig. 3). For example, glaciers in many high-latitude regions (Alaska, Greenland periphery, Russian Arctic, Svalbard and Jan Mayen, Subantarctic and Antarctic islands) account for 66% of the global glacier mass and all respond slowly to climate forcing (Fig. 3c, ‘slow response’ cluster). Mass loss continues through 2500 in these regions, and no regrowth occurs in the overshoot scenario because these relatively flat glaciers are too slow to respond (Fig. 3f–h).

Other mid- to high-latitude regions show moderate to no influence of climate overshoot (western Canada and United States, Arctic Canada, Iceland, Scandinavia and Southern Andes, with 30% of the global glacier mass) (Fig. 3d, ‘small overshoot influence’ cluster). The similarity between the scenarios is due to the limited remaining glacier mass in some regions, the dominant signal from present-day committed warming (especially in Arctic Canada South, with its substantial ice caps) and regional temperature change projections being similar between the overshoot and stabilization scenarios (Iceland).

Finally, several lower-latitude glacier regions in steep mountain areas (Central Europe, all Asian regions, low latitudes and New Zealand, with 5% of the global glacier mass) have faster response times than the idealized example of a fast-responding glacier (Fig. 1d). Their glaciers shrink considerably under the 3.0 → 1.5 °C overshoot until around 2350, but almost regrow to the 1.5 °C stabilization levels by 2500 as temperatures cool back down to the target level (Fig. 3e,g,h, ‘fast response’ cluster). Within this cluster, the rates of glacier regrowth depend on local geographic and climatic factors, and glacier regions show a wide range of responses (Fig. 3f).

## Glacier runoff response and trough water

The projected glacier runoff was aggregated to water-resource-relevant basins instead of glacier regions. We projected that glacier runoff would peak and then decline before 2100 in many basins and across all scenarios (Supplementary Figs. 3–5), consistent with previous studies^28,31,32^. However, our study assessed large-scale glacier runoff beyond 2100, where glacier runoff is projected to be permanently lower, relative to 2000–2020, in 75% of the major glaciated river basins across all scenarios because the 2000–2020 period is often near peak water. Interdecadal change in runoff varies between basins due to different glacier response times, initial states (pre- or post-peak water) and regional climate responses. The ‘fast response’ cluster (Fig. 3c) represents a small portion of the global mass, but is concentrated in mid-latitudes, in heavily populated mountain basins where glaciers are crucial to the water supply^33^. In these basins, trough water (Fig. 1c) may be a consequential temperature overshoot risk.

**Fig. 4:**
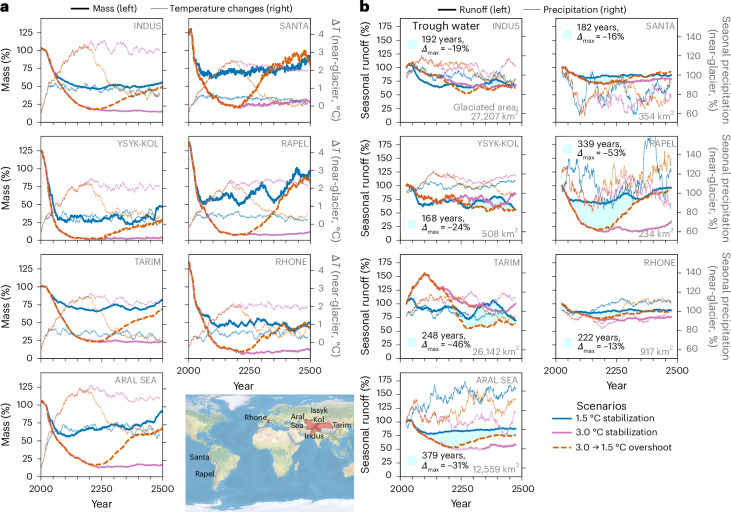
Glacier mass and dry-melt-season runoff projections for the seven relatively arid and strongly glaciated basins under the GFDL-ESM2M climate model. **a**, Glacier mass relative to 2020 and glacier-area weighted basin-wide temperature changes to 2000–2019 (both 21-year averaged). **b**, Glacier runoff relative to 2000–2050 and respective precipitation relative to 2000–2019 (both from the three driest months in the melt season and 51-year averaged). For each basin, we give the glaciated area and the number of years with estimated trough water with the maximum difference (*Δ*_max_). We give the driest melt months in Supplementary Fig. 8. We only show here three scenarios to allow for a better comparison. The same illustration for only the melt components is in Supplementary Fig. 6. The map shows the outlines of the seven selected basins. All basins have an aridity index <2 (according to ref. ^2^) and are initially above 0.9% glaciated. The projected annual glacier runoff of all 60 glaciated basins is in Supplementary Figs. 3–5. Map from Natural Earth (https://www.naturalearthdata.com/).

Dry-melt-season runoff was evaluated for seven relatively arid and heavily glaciated basins in Asia, South America and Europe that experience regular periods of low precipitation such that potential trough water may have the greatest impact (Fig. 4). The selected basins all experienced a regional temperature overshoot in the global overshoot scenarios, regaining most of their glacier mass by 2500 and forming trough water in the dry-melt season. In the most extreme case, the Rapel Basin is projected to have more than 300 years of trough water and, at its minimum, at least 50% less seasonal runoff from initially glacierised areas. Dry-melt-season trough water is more intense and persistent than annual-runoff trough water (Supplementary Figs. 3–5). For three of the basins (Indus, Ysyk-Kol and Tarim), the projected precipitation increases with rising temperature in the dry months of the melt season, which explains why the dry-melt-season runoff is greater for the 3.0 °C stabilization scenario than the 1.5 °C stabilization or 3.0 → 1.5 °C overshoot scenarios (Fig. 4b), with the annual meltwater from snow and ice melt being similar (Supplementary Fig. 6).

Overall, annual trough water occurs in 31 out of the 60 glaciated basins after 2170 (Supplementary Fig. 7). Trough water in 24 of those basins is caused by glacier mass regrowth after the temperature overshoot. For basins where precipitation decreases with warmer temperatures, longer and more intense trough water becomes more likely (consistent with the idealized experiments) (Supplementary Fig. 2).

## Discussion

Our results show that a global temperature overshoot results in substantially more glacier mass loss for decades to centuries once the cooling phase begins. The degree of temporal irreversibility is dependent on the magnitude of the overshoot, but even small overshoots will have long-lasting impacts on glacier contributions to sea-level rise (Extended Data Fig. 3). Glaciers respond variably to warming due to differences in their geometry and sensitivity, with committed mass losses dependent on the glaciers’ response times. To understand the regional water availability of glacier runoff, it is crucial to consider whether the 2000–2020 period is near peak water (for example, the basins in Central Europe) or if peak water may occur only until the twenty-second century (for example, the Tarim Basin) (Supplementary Figs. 3–5). Our results show that steeper glacier regions respond faster to overshoots (Fig. 3g), experiencing quicker mass loss initially and partial regrowth later, which aligns with findings from response-time studies (for example, ref. ^22^). Slower-responding glaciers strongly dampen the overshoot signal and reduce, or completely prevent, trough water (Fig. 1 and Extended Data Fig. 1). Furthermore, some glacier regions experience substantial warming from the overshoot (for example, the Russian Arctic, Arctic Canada North, Svalbard and Jan Mayen), while others see weaker (for example, the Southern Andes) to no (Iceland) overshoot, or ongoing regional warming despite global stabilization (for example, the Greenland periphery, Iceland, Subantarctic and Antarctic islands) (Fig. 3h and Extended Data Fig. 3). The stabilization and overshoot scenarios from the only available ESM (GFDL-ESM2M^13^) enable us to disentangle overshoot effects from changes occurring during temperature stabilization. Although idealized approaches have been developed to investigate committed and overshoot responses^34–36^, the GFDL-ESM2M simulations consist of, to our knowledge, the only approach that constructs both temperature-focused stabilization and overshoot scenarios with a historical period and realistic carbon dioxide (CO_2_), aerosol and non-CO_2_ forcings, and extends until 2500. While the physical response of the GFDL-ESM2M, particularly for temperature and precipitation patterns, falls well within the range of multi-model means^37,38^, our study shows only one potential outcome of a regional overshoot glacier response. Regional temperature and precipitation responses during overshoot and stabilization phases can differ between ESMs^12^. Consequently, the regional glacier response presented in this study may also vary depending on the ESMs used, which highlights the need for additional ESM overshoot simulations to quantify the uncertainties with our estimates. Nevertheless, other ESM stabilization studies have shown similar regional warming trends around the Subantarctic and Antarctic islands during global stabilization phases^39–41^, underscoring the potential robustness of our results.

An overshoot results in irreversible glacier change over many centuries. We use the term ‘irreversible’ as defined by the Global Tipping Points Report 2023—that is, “a change in a system that is not reversed under the same boundary conditions that triggered it, or that takes significantly longer to recover from than the time it took to reach”^19^. Our model setup using the OGGM did not include positive feedbacks, such as the climate-independent retreat of calving glaciers if destabilization thresholds are reached^42–44^, surface darkening by decreased snow cover, dust, black carbon, thin debris or glacier algae^45–47^, glacier-related slope failures or thermokarst processes^48,49^, nor negative feedbacks, such as thicker debris cover, potentially decreased ice flow^50^ or terrestrial uplifting from glacial isostatic adjustment^51^. However, our model setup accounted for the positive mass-balance elevation feedback and the negative glacier retreat to higher altitudes feedback^52^. With our model setup, we found no hysteresis in ice caps, as has been theorized for ice sheets^16^. One possible reason is OGGM’s flowline approach, which employs the shallow-ice approximation and only partially captures ice cap dynamics. However, this influence appears limited because we still projected the largest ice caps would melt away entirely under the current climate, given sufficient time^53^. Non-ice-cap-like glaciers—thus, typical mountain glaciers—can retreat to higher altitudes and regrow from there. Although the first studies including individual feedbacks in regional models indicated limited influence on regional projections (for example, debris cover^4^, calving^54^), we expect the cumulative impact of all non-accounted feedbacks to increase the temporal irreversibility of glacier mass loss beyond our current projections. Additionally, the ESM and glacier model are not coupled—that is, related feedbacks to the ocean or atmosphere are not considered. We only used one global glacier model, but qualitatively compared the model response to two other global glacier models using the projections from ref. ^55^ and one regional model^56^ under CMIP6 temperature overshoots. We observed regrowth in fast-responding regions, but the regrowth magnitude up to 2300 varies greatly across glacier models (Supplementary Fig. 1). The different temperature overshoots and lack of comparative stabilization scenarios in CMIP6 prevent the quantification of uncertainty from ESMs and further analysis of overshoot glacier impacts, such as sea-level rise or trough water (Methods).

We have introduced the concept of trough water for glacier runoff in temperature overshoot and stabilization scenarios. This projected reduced glacier runoff occurs specifically in steeper fast-responding non-Arctic basins when glaciers regrow. However, an overshoot scenario also impacts the adaptation planning of near-future water availability, with peak water being more intense and shorter under the overshoot scenario, even for basins without trough water. How relevant a potential glacier-runoff trough water might be for river basin discharge downstream in periods of drought or low precipitation depends on many aspects of the discharge regime. The different non-glacierised components of mountain catchments (for example, snow, vegetation, permafrost and groundwater storage) and non-accounted glacierised components (for example, debris cover) buffer runoff on different time scales, and glacier runoff is often only a small contributor to the entire runoff downstream^57^.

Future studies need to assess whether, and how far downstream, the signal of glacier-runoff trough water remains important when forcing temperature overshoot scenarios on coupled glacier–hydro large-scale modelling approaches. Coupling large-scale glacier models with hydrological models shows that glaciers alter future discharge^32^, so representing glaciers explicitly is important in hydrological models^58^. For that, absolute glacier runoff contributions are necessary, despite their large uncertainties^31^. Separating glacier-sourced runoff ablation components (here approximated by the glacier meltwater components) into a balanced (glacier mass compensated by accumulation on an annual basis) and imbalanced (present-day committed loss) term could give further insights^57,59^.

The world after an overshoot will be different from the world before the overshoot^12^. Glacier impacts on future water availability and sea-level rise are just two examples of the uncertain dangers resulting from delayed action. Surpassing 1.5 °C global warming necessitates returning to, or below, 1.5 °C to avoid long-term tipping points in the climate system^15,18,19^. Counting on climate mitigation by overshoot from cooling temperatures over the coming centuries is risky due to physical climate system feedbacks, uncertainties on the scalability of CO_2_ removal techniques, and the nonlinear dependence of glacier response on the temporal pattern of mitigation^12,60^. Paradoxically, having reached 3.0 °C global warming, some basins’ glacier runoff might be reduced more under global cooling than if the climate were held steady there because of glacier regrowth. Cooler temperatures might be locally detrimental, raising fears of trough water in glacier-dependent sectors. Trough water therefore puts local climate adaptation interests in tension with global climate mitigation interests. Relying on future carbon reductions rather than taking action in this decade will have unforeseen consequences, including local disputes over cooling impacts. Immediate emissions reductions are essential to avoid competing motivations in a post-overshoot world. This study underscores the urgency of near-term emissions reductions to limit peak global warming to the lowest possible level.

## Methods

### Glacier model

We simulated the mass and runoff of more than 200,000 individual mountain glaciers using the open-source numerical modelling framework OGGM^27^, which has been employed in several global and regional mass change studies^54,61^ as well as in hydrological studies^31,32,62,63^ (as the most recent examples). For this study, we used the default configuration of OGGM, v.1.6.1 (ref. ^64^), as described in ref. ^61^ and on the OGGM documentation website (https://docs.oggm.org/en/v1.6.1/). The Randolph Glacier Inventory (RGIv6.0)^65^, elevation-band flowlines^66,67^ and an informed three-step dynamic calibration were applied to calibrate the monthly temperature index model to the geodetic glacier-wide mass-balance measurements for each glacier^68^ within 20% of their error estimate. The applied dynamical spinup matches the RGI area for every glacier at the RGI inventory date, and the glacier volume of the current multi-model estimates^69^ is matched regionally. The glacier density was assumed to be 900 kg m^−3^ to convert it to mass.

### Glacier model simulation

For 2000 to 2019, we simulated glacier mass and runoff using the OGGM forced by the W5E5v2.0 climate dataset^70^. For 2020 through 2500 (and beyond for extended stabilization and overshoot scenarios over a total of 10,000 years), we projected glacier changes forced using output from GFDL-ESM2M for five stabilization and three overshoot scenarios (see below). The projected air temperature and precipitation were bias-corrected to the period 1980–2019 using additive and multiplicative factors, respectively, as described in ref. ^67^. Results are shown for 98.7% of the glaciers (99.6% by area) that successfully ran for all scenarios—that is, we excluded glaciers with insufficient initialization data or simulations that failed when the glacier area exceeded the model boundaries in at least one scenario before 2500.

To convert global glacier mass changes to sea-level rise contributions (Fig. 2d), we used the above-sea-level volume estimates from the OGGM and assumed an ocean area of 3.625 × 10^8^ km^2^, an ocean water density of 1,028 kg m^−3^ and a glacier density of 900 kg m^−3^ (ref. ^71^). Glacier mass lost below sea level already displaces ocean volume and does not substantially contribute to sea-level rise when it melts. Therefore, we omitted the below-sea-level volume estimates from the global mass loss estimates before converting the changes to sea-level rise estimates. As is commonly done^72^, we assumed all meltwater from glacier mass loss immediately reaches the oceans.

Runoff was estimated as the sum of melt and liquid precipitation using a ‘fixed-gauge’ approach based on the glacier area in 2000—that is, snow melt and liquid precipitation from non-glaciated areas is included in the glacier runoff as the glacier retreats (Extended Data Fig. 1n–s). If the glacier grows beyond the initial area during the simulation, the runoff is taken from that larger area during those years. This occurred in six small basins after 2020 and only for single years. The impact on the results is thus negligible in the basin-scale trend analysis. In this study, we report centred rolling mean runoff trend patterns over 21 years for the idealized 21-year repeated climate experiments (Fig. 1, Extended Data Fig. 1 and Supplementary Fig. 2) and over 51 years for the real-world basins forced by one ESM (Fig. 4 and Supplementary Figs. 3–7) due to the larger interannual precipitation variability in the ESM simulations. Dry-melt-season glacier runoff was estimated for the three-month period within the melt season that had the lowest monthly W5E5v2.0 precipitation in 1990–2019 for each basin or glacier (Fig. 4, Extended Data Fig. 1 and Supplementary Fig. 8). The three-month period precipitation seasonality was computed using a three-month rolling mean basin precipitation, which was then averaged over the 30-year period for each month. Subsequently, the six-month period with the highest melt within a year was estimated from the glacier simulations (2000–2019), during which the driest three-month period was selected. This dry period was based on past meltwater and precipitation climatology and does not account for potential climate-change-induced shifts in precipitation or melt seasonality.

We defined trough water to occur if the 21- or 51-year average annual or seasonal glacier runoff from an overshoot scenario is at least 5% smaller than in a stabilization scenario for at least 20 years and also 5% smaller than in the baseline period (initial steady state for the idealized experiments, 2000–2020 or 2000–2050 climate for the projections from the ESM).

### Glacier aggregated climate, mass and runoff projections

The glacier projections were regionally aggregated for analysis. The glacier mass was aggregated to the 19 regions of the RGIv6.0, with glacier runoff aggregated to the 60 Global Runoff Data Centre (GRDC)^73^ major river basins with at least 0.1% glacier cover around 2000. These 60 basins are all outside Antarctica and Greenland, have at least 30 km^2^ of glacier cover and are at least 3,300 km^2^. Glaciers were mapped onto basins based on their central latitude and longitude. Global, regional and basin climate and glacier characteristics, such as glacier surface slopes, were aggregated using area-weighted averages based on the area associated with RGIv6.0. For example, regional glacier climate (as shown in Figs. 2b and 4a, Extended Data Fig. 3 and Supplementary Figs. 3–6 and 9) was determined by applying an area-weighted average to the non-bias-corrected annual temperature and precipitation from the nearest ESM gridpoint for each glacier.

A cluster analysis was performed on the mass differences from 2000–2500 between the 3.0 → 1.5 °C overshoot and 1.5 °C stabilization scenarios for the 19 glacier regions to identify regions with a common response in how they differed between the two scenarios. The mass differences were normalized relative to the 2020 mass before *k*-means clustering^74^. We evaluated different cluster numbers and found that four clusters distinguished the regions well while remaining interpretable. When more than three clusters were used, the Caucasus and Middle East region always formed their own cluster because this had the largest overshoot influence, but also the largest and fastest regrowth (Extended Data Fig. 3). We thus manually merged this region with the 'fast response' cluster (those regions being less extreme) (Fig. 3c). Given that the Caucasus and Middle East region had the smallest initial mass in 2020 (0.03% of global), qualitatively, this region remained well represented.

Additional regional and basin-wide analyses of these supplementary figures is presented in the Supplementary Information.

### Idealized experiments on fast- and slow-responding glaciers

Idealized experiments, in which we specified temperature and precipitation changes, were conducted for the largest glaciers in six regions (Central Europe, Central Asia, low latitudes, Alaska, Arctic Canada North and Arctic Canada South) to support our interpretations of regional changes in the stabilization and overshoot scenarios. For these idealized experiments (Fig. 1, Extended Data Fig. 1 and Supplementary Fig. 2), we applied the 1999–2019 average climate with a time-dependent temperature adjustment mimicking the overshoot or stabilization scenarios. We added a glacier-specific temperature adjustment (for example, −1.15 °C for Aletsch Glacier in Central Europe) to the 21-year 1999–2019 climate to ensure the experiment began with the glacier in a steady state, with approximately the same glacier mass as in 2000.

We performed one idealized linear increase and one overshoot experiment that reached the same temperature level after 250 years, after which the temperature was held constant (Fig. 1a). The linear increase experiment increased the temperature linearly to +0.6 °C over 136 years. The idealized overshoot experiment increased the temperature by +2.0 °C and then reduced the temperature back down to +0.6 °C over 250 years. The overshoot experiment peaked in the year when the stabilization experiment stabilized. Precipitation was kept constant. Runoff was shown as a 21-year centred rolling mean to smooth interannual changes. Model simulations were run for up to 4,000 years to ensure the glaciers returned to a new steady state.

Extended Data Fig. 1 extends the idealized experiments to the largest glaciers from six glacier regions. For every glacier, a temperature bias, *t*_ss_, on top of the 1999–2019 climate was found where the glacier, after many centuries, reached a steady state with a mass similar to the glacier mass at the inventory date (around 2000). From this state, the temperature changes from Extended Data Fig. 1a were applied to every glacier, which resulted in different responses of glacier mass and runoff. The starting and ending temperatures were the same for the stabilization and overshoot experiments, and the glaciers were in, or reached, the steady state, respectively. In addition to the idealized experiments described in the Methods, simulations were performed increasing or decreasing precipitation linearly in relation to the temperature changes to assess the influence of precipitation changes on these idealized experiments (±5% °C^−1^) (Supplementary Fig. 2).

The Extended Data Fig. 1 and Supplementary Fig. 2 showing the idealized experiments are further analysed in the Supplementary Information.

### Stabilization and overshoot scenarios from GFDL-ESM2M

The glacier model was forced with monthly-mean temperature and precipitation data from eight simulations using the fully coupled GFDL-ESM2M^24,25^. The GFDL-ESM2M consists of atmospheric, oceanic, terrestrial and sea ice components, with a nominal atmospheric horizontal resolution of 2 × 2.5^∘^ and a nominal ocean horizontal resolution of 1^∘^. To conduct a set of temperature stabilization and overshoot scenarios, the model was forced with historical CO_2_, non-CO_2_ greenhouse gas (GHG) emissions, aerosols and land-use change over the period 1861–2025. After 2025, the model was forced with fossil fuel CO_2_ emissions obtained from the adaptive emissions reduction approach^26^, which allows any global mean surface warming target^26,75^ to be reached by re-evaluating the remaining CO_2_ forcing equivalent emissions budget every five years. Non-CO_2_ GHGs, aerosols and land-use change followed the low-emissions, high-mitigation Representative Concentration Pathway (RCP) scenario RCP2.6 from 2006 to 2100^76^, and were kept constant afterwards.

For Figs. 1–4, we used up to four scenarios, including three global temperature stabilization scenarios (at 1.2, 1.5 and 3.0 °C warming above pre-industrial ESM levels) and one temporal temperature overshoot scenario that peaked at 3.0 °C and then declined back to 1.5 °C (ref. ^13^). The stabilization scenario for 1.5 °C reaches this level in 2054. In the overshoot scenario, warming peaks at 3.0 °C in 2162, converging back to the 1.5 °C stabilization level in 2374.

We also forced the OGGM with four additional GFDL-ESM2M scenarios—two additional global temperature stabilization scenarios (2.0 and 2.5 °C warming above pre-industrial ESM levels) and two temporal temperature overshoot scenarios that peaked at 2.0 and 2.5 °C, and then declined back to 1.5 °C (ref. ^13^). For clarity, our figures do not show the 2.0 and 2.5 °C stabilization scenarios, but that data is available (ʽData availabilityʼ and ʽCode availabilityʼ sections). The 2.0 → 1.5 °C overshoot and 2.5 → 1.5 °C overshoot scenarios are only shown in Extended Data Fig. 3. In the overshoot scenarios, the temperature peaked at 2.0 °C in 2088 and at 2.5 °C in 2147. These temporary overshoot simulations converge back to 1.5 °C stabilization levels in 2270 and 2363, respectively.

We present the ESM temperature changes in Figs. 2 and 4 and Extended Data Fig. 3 using the IPCC AR6 definition^1^ (that is, assuming global warming levels of 0.69 °C between pre-industrial (1850–1900) times and 1986–2005^77^). This is a historical warming very close to the actual GFDL-ESM2M warming level of +0.70 °C for between 1861–1900 and 1986–2005. Because the global warming level reached 1.2 °C above pre-industrial levels in the period 2014–2023^7^, the glacier projections under the 1.2 °C stabilization scenario quantified the present-day committed glacier mass loss.

In addition, we simulated glacier evolution post-2500 using the glacier states from 2500 for the 1.5 °C stabilization and 3.0 → 1.5 °C overshoot scenarios, and repeatedly exposing these to the 2399–2499 climate from the 1.5 °C stabilization scenario over a total of 10,000 years (see more in Extended Data Fig. 2 and Supplementary Information). The results for these extended stabilization and overshoot simulations are shown for 96% of the total glacier area. The additional missing glacier area comes specifically from RGI regions 03, 07, 09, 10 and 19, in which 5–20% of the glaciers (by area) fail (that is, the glacier area exceeds the domain boundary after 2500).

### Treatment of uncertainties

The GFDL-ESM2M climate model projections^13^ used here offer a unique opportunity to explore the influence of temperature overshoots. However, this uniqueness also limited our ability to evaluate structural climate model uncertainty in the glacier projections after overshoot^5,31,72^. Future research should leverage outputs from multiple climate models, as they become available, to better quantify uncertainties in glacier projections following temperature overshoots. Given this limitation, we did not present any uncertainty estimates. However, our glacier projections show one out of many potential outcomes, which is a step beyond the idealized experiments presented (Fig. 1). Including partial uncertainties, such as those related to initial glacier volume, parameter equifinality or mass-balance observations, or adding speculative uncertainty ranges would create a misleading impression of precision. Instead, we discuss some of the uncertainties qualitatively, including the bias correction period, glacier model choice and climate model choice.

One important uncertainty source in glacier projections is the climate models’ bias correction period. For a sensitivity test, we used an alternative bias correction period (2000–2019), instead of 1980–2019, and repeated the OGGM projections under the GFDL-ESM2M scenarios up to 2500 (Supplementary Fig. 10). We found that using the bias correction period 2000–2019 resulted in 8% less glacier mass in 2500 for most scenarios than using the bias correction period 1980–2019 (default approach in our study). However, the differences between the temperature overshoot and the stabilization scenario projections in 2500 are similar (10.5 versus 11.3%) for the two bias correction periods.

To evaluate other glacier models’ responses to overshoot, we selected seven CMIP6 projections with a considerable temporal temperature overshoot (more than 0.6 °C) up to 2300, which were available for three glacier models (OGGM, PyGEM-OGGM and GloGEM) (Supplementary Fig. 1). The glacier models show a qualitatively similar global glacier mass response up to 2300, but regional responses can be substantially different between them. The differences arise from varying representations of glacier dynamics, such as the shallow-ice approximation used in the OGGM and PyGEM-OGGM versus the mass redistribution curves in GloGEM, which are less realistic under regrowth conditions^78^. Comparing temperature overshoot projections up to 2300 from GloGEM to those from a modified version (GloGEMflow^67^) with the glacier dynamics represented (GloGEMflow, only available in Central Europe for three climate models from Fig. S3 in ref. ^56^) equally shows that including ice dynamics generally leads to less glacier mass regrowth. In addition, the choices of the glacier mass balance model, calibration and baseline climate substantially influence glacier mass and runoff estimates^31,62,79^.

Differences between the climate models themselves substantially influence the glacier and runoff responses under a global temperature overshoot. It is not straightforward to compare the different climate models’ responses up to 2300 because the CMIP6 shared socioeconomic pathways result in different peak temperatures and overshoot magnitudes due to their varying transient climate responses to emissions (Supplementary Fig. 1). Nonetheless, climate models with similar global mean temperatures show glacier projections within 20% of each other in 2300, although the differences are much larger regionally because they depend on the regional temperature distribution^12^. While the regional uncertainty is not quantifiable using a single ESM, as used in our study, the CMIP6 simulations up to 2300 provide a rough estimate of the order of magnitude of the global and regional uncertainties that can be expected due to differences in the ESMs and glacier models.

The Glacier Model Intercomparison Poject Phase 2 (ref. ^72^) found that uncertainties in glacier mass projections for 2100 are equally influenced by the choice of glacier models and climate models. However, it remains unclear whether glacier model or climate model uncertainties dominate under long-term temperature overshoot scenarios. Recent advances, such as the homogeneous use of per-glacier geodetic mass-balance observations for calibration^68^, potentially reduce the spread in glacier model projections. By contrast, glacier model uncertainties arising from climate models are usually assessed under equal shared socioeconomic pathways or RCPs^61,72^ or within relatively broad global warming ranges (for example, ±0.25 or ±0.5 °C)^5^. Thus, climate-model-induced glacier mass projection uncertainties from regional temperature and precipitation patterns could diminish when the climate models all converge on the same global mean temperature target. Sea-level rise contributions from glaciers are more uncertain than relative glacier mass estimates due to the stronger dependence on the assumed initial glacier mass and mass above sea level. Glacier runoff projections strongly depend on precipitation, which, in absolute values, depends strongly on the chosen precipitation baseline and adjustment of the glacier model. However, the climate model choice may dominate over the relative glacier runoff uncertainties (for example, the year of peak water^31^).

Given the policy relevance of these overshoot and stabilization scenarios, ideally, more ESMs will produce such data in the future. Global glacier models have to improve their mechanisms for glacier regrowth. Currently, only three global glacier models with simplified ice dynamics exist—GloGEMflow^67^, OGGM^27^ and PyGEM-OGGM^5^. For a comprehensive and robust quantitative uncertainty analysis, future overshoot studies should build on this work by employing multiple climate models with overshoot scenarios across as many independent glacier models as possible while also assessing uncertainties from glacier observations and model internal processes.

## Online content

Any methods, additional references, Nature Portfolio reporting summaries, source data, extended data, supplementary information, acknowledgements, peer review information; details of author contributions and competing interests; and statements of data and code availability are available at 10.1038/s41558-025-02318-w.

## Supplementary information

Supplementary Information Supplementary Figs. 1–10 and Discussion.

## Data Availability

The overshoot and stabilization OGGM glacier projection data under GFDL-ESM2M (from 2000 to 2500, and the extended experiments presented in Extended Data Fig. 2) and the glacier-region extracted climate data are available via Zenodo at 10.5281/zenodo.14247718 (ref. ^80^), documented in README_data.md. Glacier projection data up to 2300 from the CMIP5 and CMIP6 climate models and from three glacier models (for Supplementary Fig. 1) are available via Zenodo at 10.5281/zenodo.10055416 (ref. ^55^).

## Code availability

Code for the climate preprocessing, OGGM projections, postprocessing, figure and additional analyses is available via Zenodo at 10.5281/zenodo.14247718 (ref. ^80^), documented in README.md. The OGGM code (v.1.6.1) used is available via Zenodo at 10.5281/zenodo.8287580 (ref. ^64^).
