# Supplementary material for: Irreversible glacier change and trough water for centuries after overshooting 1.5 °C
Source: Nat Clim Chang. 2025 May 19;15(6):634–41. doi: 10.1038/s41558-025-02318-w (PMC12158768; doi:10.1038/s41558-025-02318-w)
Supplement: Supplementary file 1 — Supplementary Figs. 1–10 and Discussion. [file 41558_2025_2318_MOESM1_ESM.pdf]

nature climate change

Supplementary information

https://doi.org/10.1038/s41558-025-02318-w

Irreversible glacier change and trough water for centuries after overshooting 1.5 °C

In the format provided by the authors and unedited

Table of Contents

Comparison to other glacier mass projections under global temperature overshoots 2

Additional idealised experiments 3

Glacier mass projections under GFDL-ESM2M extended scenarios 5

Additional discussion on global and regional glacier mass projections under GFDL-ESM2M . . 6

Additional glacier runoff projections under GFDL-ESM2M 6

Additional analysis on local or regional climate of W5E5 or GFDL-ESM2M 12

Analysis of the bias correction period influence on glacier mass projections 13

Comparison to other glacier mass projections under global temperature overshoots

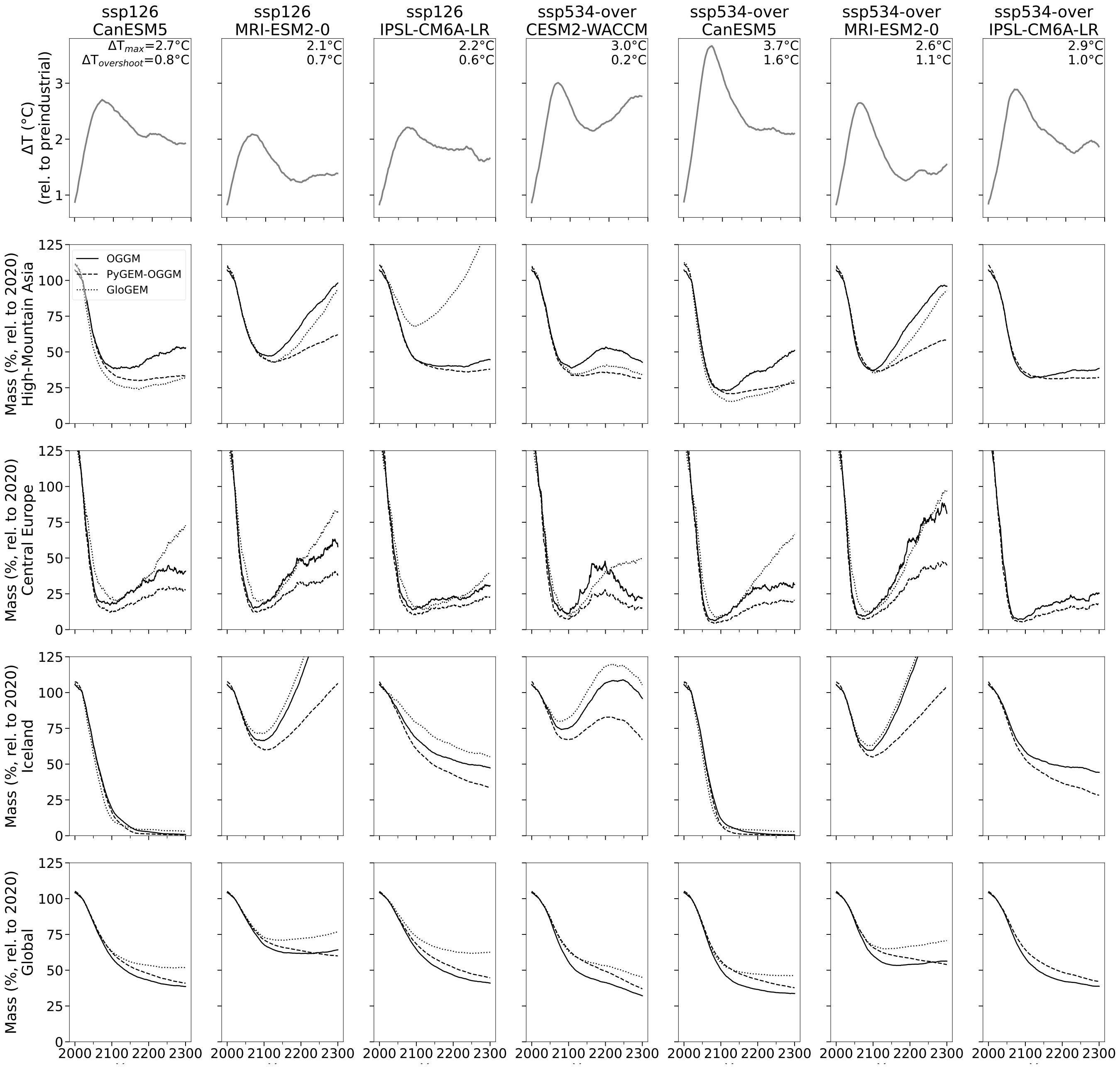

Supplementary Figure 1. **Global mean temperature change ΔT (31-year averaged) and regional and global glacier mass projections until year 2300 with CMIP6 under the seven Shared Socio-economic Pathways (SSPs) and climate model combinations that show a considerable temperature overshoot (>0.6°C)**. Maximum ΔT during overshoot (ΔT_{max}) and difference to the minimum ΔT after the peak ($\Delta T_{overshoot}$) are shown. Mass changes are shown in % relative to 2020 in High-Mountain Asia (containing the RGI regions Central Asia, South Asia West and South Asia East), Central Europe, Iceland and globally for the three different large-scale glacier models (data from ref.[1]). The available glacier simulations come from the glacier model OGGM (same version as for the GFDL-ESM2M projections, i.e., OGGM v1.6.1, but with bias correction from the period 2000–2019; original model ref.[2]), PyGEM-OGGM (similar version as in[3]), and GloGEM (version as in[4]; original model ref.[5]). GloGEM projections were not available for IPSL-CM6A-LR SSP5-3.4-over.

Additional idealised experiments

In the following, we provide further details on the idealised experiments presented in Fig. 1, and additionally analyse the idealised response of other glaciers. This extended analysis helps us better understand the regional variability of glacier mass and runoff responses to climate projections.

Annual runoff reaches the same level as in the previous steady state since the contributing area and precipitation do not change. However, the runoff components and seasonality do change (Extended Data Fig. 1), which is counterbalanced in the other months by increased runoff due to increased liquid precipitation and off-glacier snow melt. With this idealised model experiment, we could reproduce with OGGM the schematic illustration of peak water of Huss & Hock[6]. However, our model-based experiments also show the time-lagged response of glacier mass and annual and seasonal runoff, which is important for slow-responding glaciers. This time-lagged response is not visible in Huss & Hock[6].

Slow-responding glaciers show a less intense and more stretched (longer) period of trough water (Extended Data Fig. 1). For glaciers that respond even slower than the temperature overshoot recovery (e.g. Wykeham Glacier South or Barnes Ice Cap), no regrowth occurs, and thus, no trough water occurs. Note that the melt components of, e.g., Wykeham Glacier South are very sensitive to temperature changes and liquid precipitation components from the runoff are small, which explains the doubling of the runoff. For most glaciers, the runoff response of the driest months within the melt season follows roughly the shape of the annual response. However, our idealised experiments started with glaciers being in a steady state with the climate; in reality, glaciers are not in balance and, e.g., peak water might have already occurred.

We analyse idealised precipitation changes experiments to better understand the influence of precipitation changes on glacier mass and runoff projections (Supplementary Fig. 2). In general, with increasing temperatures, precipitation often increases but can regionally also decrease. If precipitation increases with temperature, glacier mass loss is reduced, the peak water is slightly more intense and delayed, and the trough water in the overshoot experiment is also less intense and shorter. If precipitation decreases while the temperature increases, the peak water occurs earlier and is less intense; conversely, trough water starts earlier, is slightly more intense, and lasts longer.

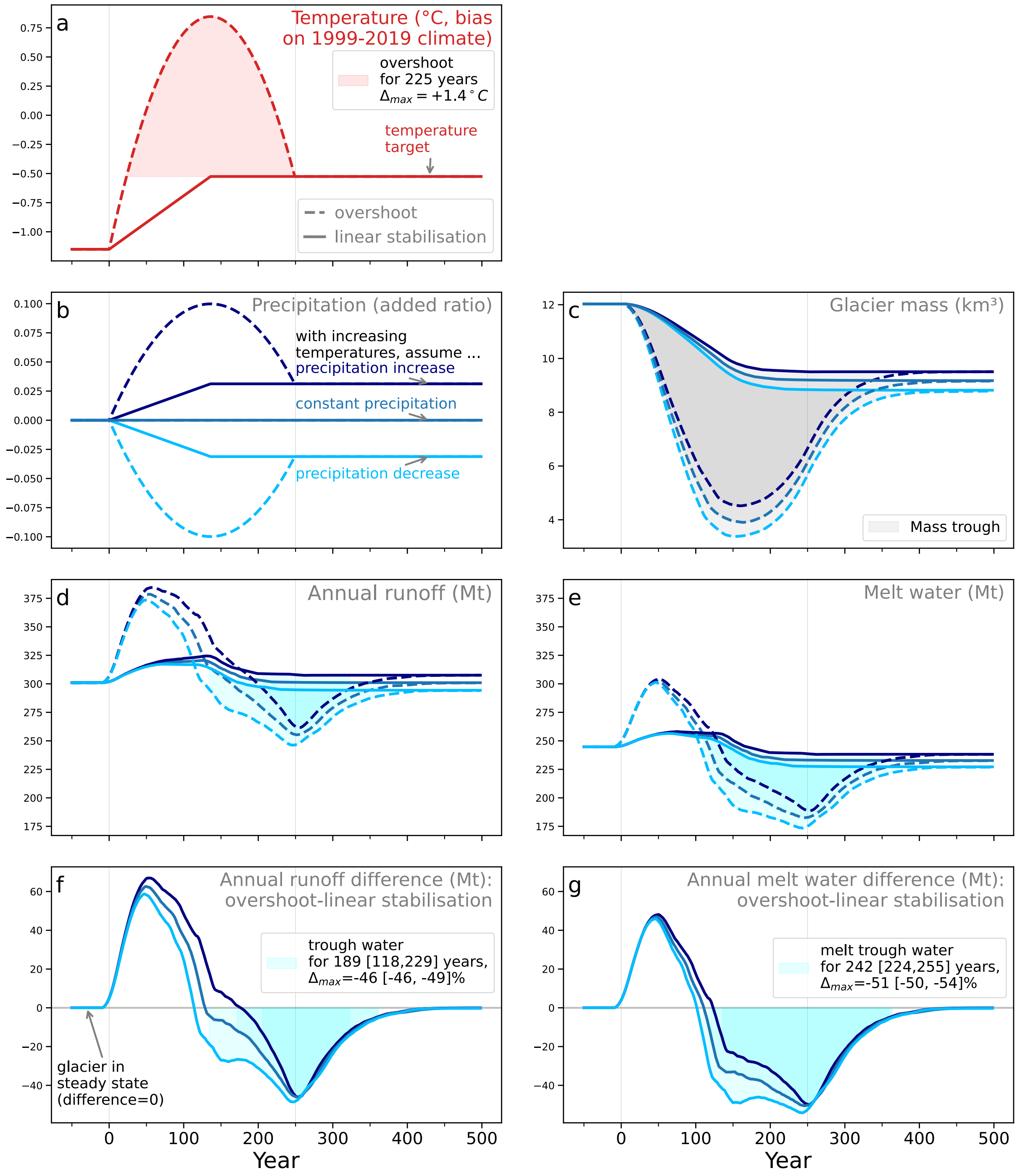

Supplementary Figure 2. Influence of precipitation changes on glacier mass and annual runoff changes under an idealised stabilisation and overshoot experiment with the fast-responding Aletsch glacier. **(a)** We applied the same temperature changes as in Fig. 1 and Extended Data Fig. 1. **(b)** The precipitation changes were increased, kept constant or decreased linearly for a temperature increase ($\pm 5\%\ °C^{-1}$). **(c)** shows the precipitation-change-dependent mass changes. The mass differences between the overshoot and the linear stabilisation experiment ("Mass trough") are highlighted. **(d, f)** shows the annual runoff and annual runoff differences between experiments (both 21-year averaged). Annual glacier-runoff trough water is highlighted. The number of years with trough water and the maximum runoff depletion (Δ_{max}) for the different precipitation change experiments are mentioned. **(e, g)** show only the glacier meltwater components of the glacier runoff (21-year averaged), i.e., excluding the liquid precipitation components within and outside the glacier area.

Glacier mass projections under GFDL-ESM2M extended scenarios

The scenarios until 2500 reveal large parts but not the complete committed glacier mass losses due to the slow response of some flat, large glacier regions. Our extended stabilisation and overshoot projections show that glaciers continue losing mass beyond 2500 before finally reaching a new steady state (Extended Data Fig. 2). While global glacier loss converges to similar steady states in the millennia following the applied overshoot, it remains globally irreversible relative to the stabilisation scenario for centuries post-overshoot.

Note that there are more failing glaciers for the over 10,000 year-long projections, which explains why the differences between the 1.5°C Stabilisation and the 3.0→1.5°C Overshoot are 10.4% in the year 2500 under the non-failing glaciers of the extended projection, while they are 11.3% when accounting for all non-failing glaciers until 2500 (as shown in Fig. 2b). In addition, some marine-terminating glaciers in the Subantarctic & Antarctic Islands do not fully converge to a similar steady state in the two extended scenarios even after 10,000 years of equal climate. The reasons are unclear and require analysis of potential glacier model dependence. Furthermore, we assumed that for our extended stabilisation and overshoot scenarios, the local climate after the overshoot would be the same as for the stabilisation scenario. However, global climate reversal does not imply local climate reversal, even over extended periods[7]. Glaciers in Greenland Periphery, Iceland and Scandinavia, for example, may be impacted by a delayed response of Atlantic Meridional Overturning Circulation feedbacks[7,8]. Glaciers in continental regions may be affected by the stronger cooling over land[9]. Conversely, glacier regions near high present-day aerosol loading may warm up more due to future aerosol reduction. Thus, local glacier states will remain distinct due to incomplete reversal of local climates even after global temperature reversal.

Additional discussion on global and regional glacier mass projections under GFDL-ESM2M

Compared to Fig. 3, we show in Extended Data Fig. 3 the individual RGI region glacier mass response and, in addition, weaker overshoots to demonstrate the magnitude's sensitivity. Glaciers globally are projected to lose 30 to 62% of their mass by 2500, relative to 2020, for the 1.2 to 3.0°C Stabilisation scenarios. The overshoots peaking at 2.0°C or 2.5°C in 2500 result in 3% or 9% more global glacier mass loss compared to the stabilisation scenario while the overshoot peaking at 3.0°C results in 11% more mass loss in 2500.

The cooling after peak warming and the glaciers' response times are highly heterogeneous and thus the impacts are region-specific. Within the fast-responding regions, glacier regions regrow at a different pace; e.g. Central Europe regrows completely to the 1.5°C Stabilisation scenario mass level until 2500, while for Central Asia, the glacier mass is still 14% smaller until 2500 for the 3.0→1.5°C Overshoot compared to the 1.5°C Stabilisation scenario. Within the cluster of the slow-responding regions, the signal from Subantarctic and Antarctic Islands (30% of global mass) with a zero regrowth dominates the cluster signal in Fig. 3c, overshadowing the smaller glacier mass regions of that cluster, Alaska (01, 12% of global mass) and Svalbard & Jan Mayen (07, 5% of global mass) with small, but notable regrowth.

The regional glacier mass response to a global temperature overshoot is similar to our idealised experiments with glaciers of different glacier geometries (Extended Data Fig. 1). However, unlike the idealised experiments, the projections start with glaciers that are not in a steady state with the current climate. In some regions (mainly Arctic Canada North and South), small projection differences occur despite large regional temperature differences because the committed mass loss at 1.2°C dominates the response until 2500 (Extended Data Fig. 3). Specifically, Arctic Canada South has many slow-responding ice caps (such as the Barnes Ice Cap[10]). For Scandinavia, W Canada & US, and Arctic Canada South less than 25% glacier mass is projected to remain until 2500 under the 1.5°C Stabilisation scenario (Fig. 3f) scenario, which limits potential differences for the warmer 3.0→1.5°C Overshoot scenario. In addition, regional climate changes are different to the global response. For example, in Iceland, the projections differ little because the regional temperature change is similar between the scenarios in the GFDL-ESM2M. Also note that interdecadal near-glacier projected precipitation variability (Supplementary Figs. 3-5) might explain some of the projected interdecadal glacier mass changes.

Additional glacier runoff projections under GFDL-ESM2M

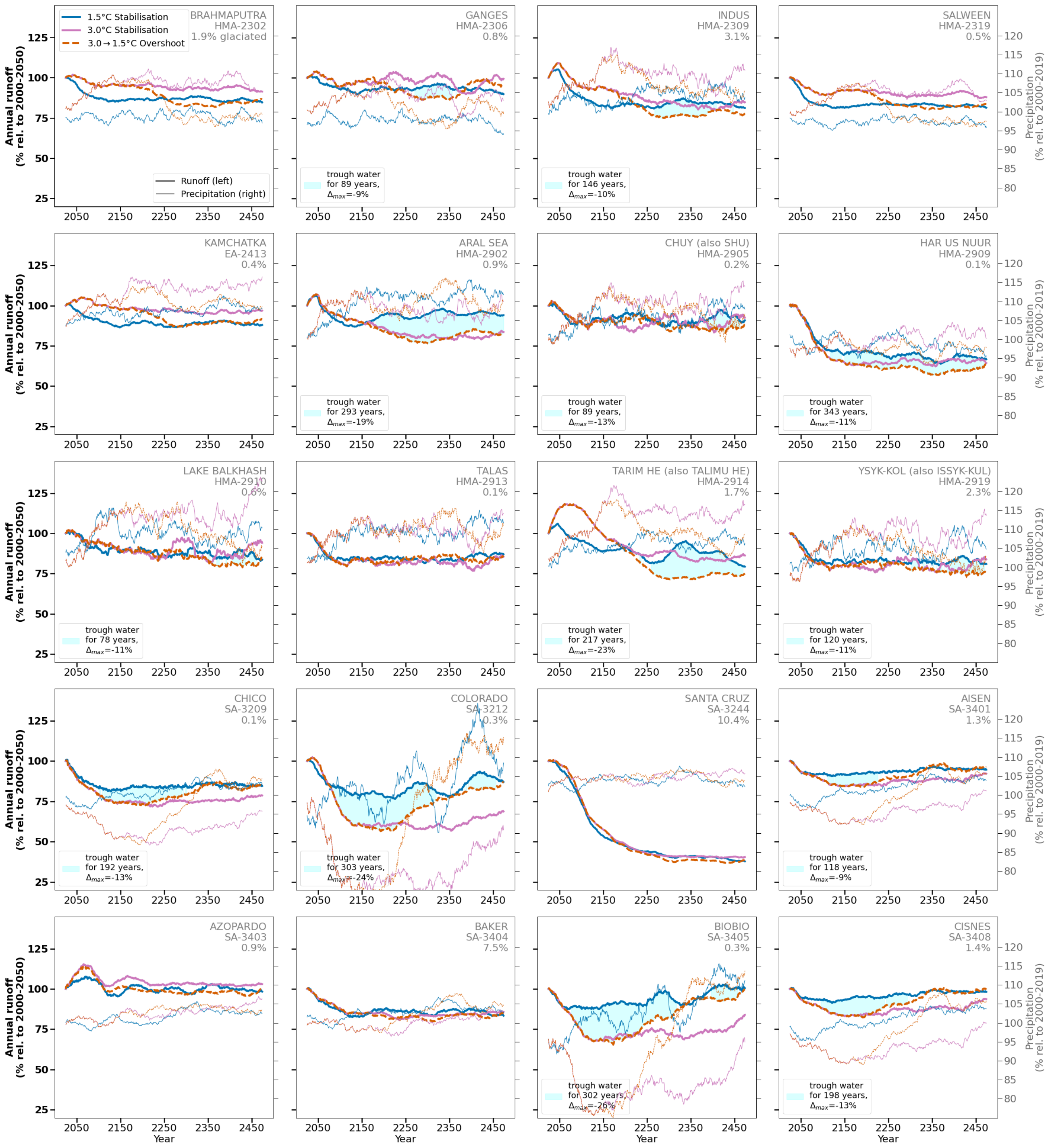

Supplementary Figure 3. **Annual glacier runoff projections (relative to 2000–2050) and respective near-glacier precipitation (relative to 2000–2019, both 51-year averaged)**. We show the first 20 glaciated basins, sorted by basin index. The basins shown are located in High-Mountain Asia (HMA) and the Southern Andes (SA); with the basin name, region, and Global Runoff Data Centre (GRDC) index provided. For each basin, the glaciation extent around year 2000, the number of years with estimated trough water and the maximum difference (Δ_{max}) are indicated. Only three scenarios are presented to enhance comparison.

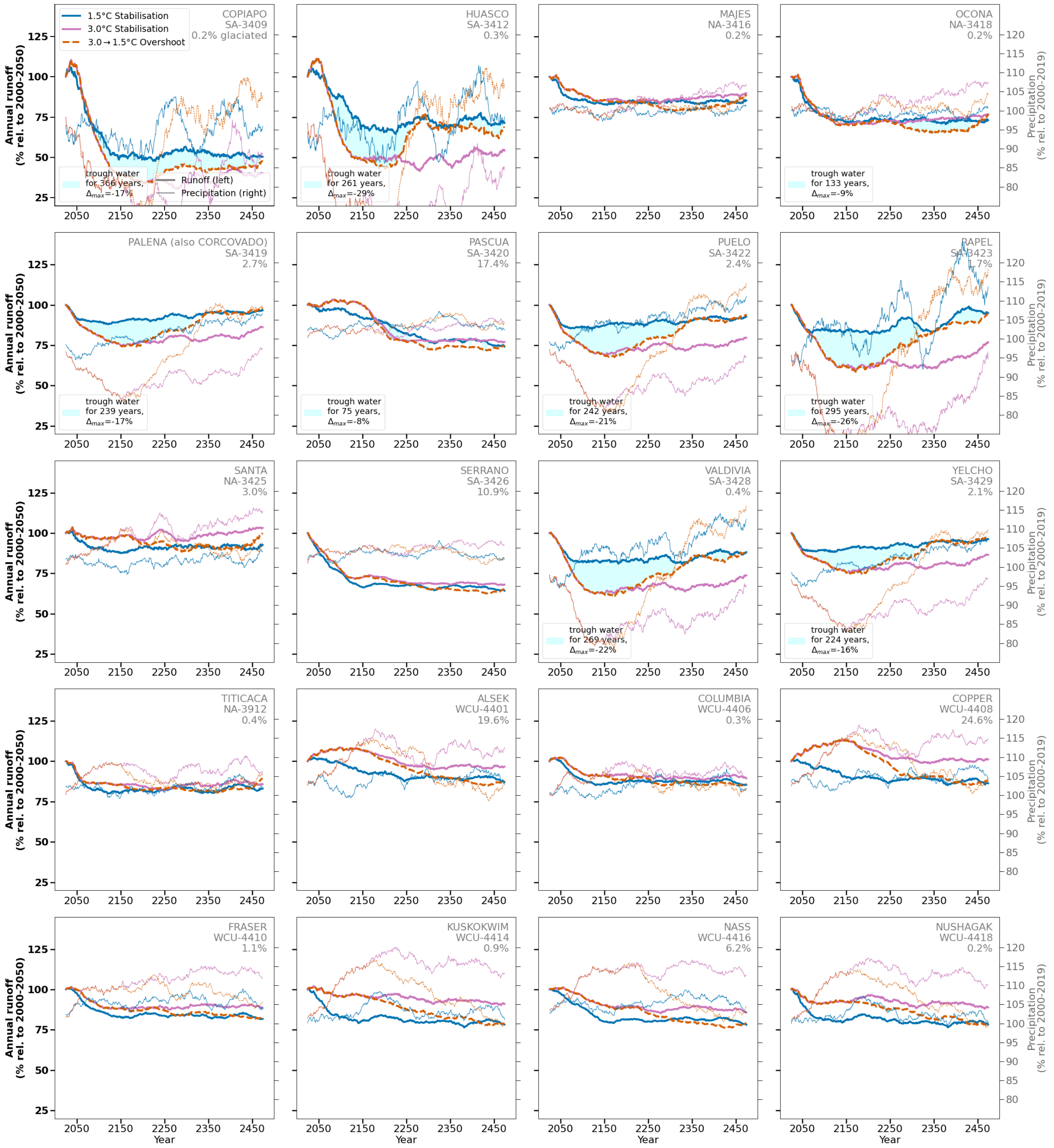

Supplementary Figure 4. **Annual glacier runoff projections (relative to 2000–2050) and respective near-glacier precipitation (relative to 2000–2019, both 51-year averaged)**. We show the next 20 glaciated basins following those presented in Fig. 3, sorted by basin index. The basins shown are located in the Southern Andes (SA), Northern Andes (NA), and Western Canada & USA (WCU); with the basin name, region, and Global Runoff Data Centre (GRDC) index provided. For each basin, the glaciation extent around year 2000, the number of years with estimated trough water and the maximum difference (Δ_{max}) are indicated. Only three scenarios are presented to enhance comparison.

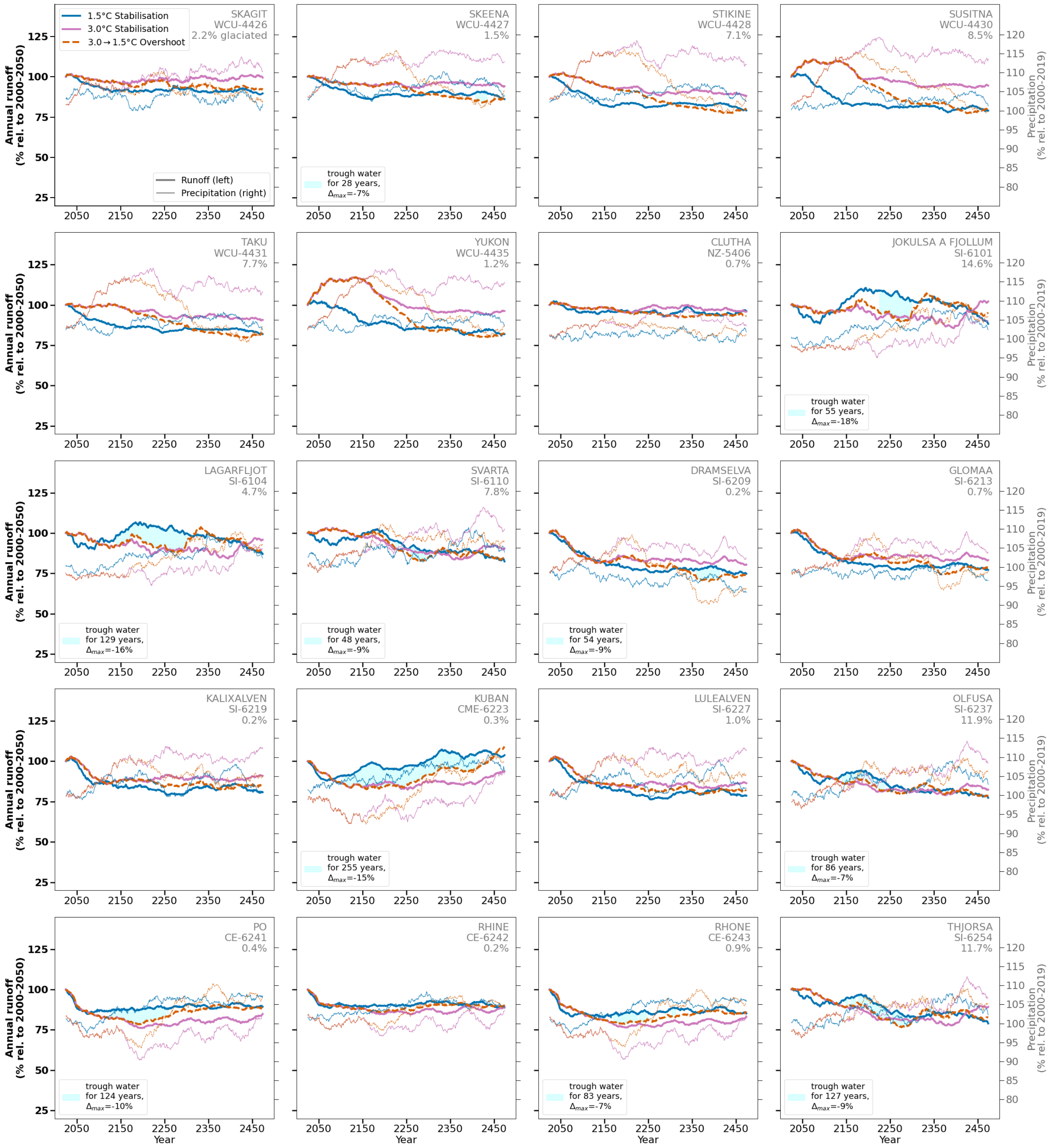

Supplementary Figure 5. **Annual glacier runoff projections (relative to 2000–2050) and respective near-glacier precipitation (relative to 2000–2019, both 51-year averaged)**. We show the last 20 glaciated basins following those presented in Fig. 4, sorted by basin index. The basins shown are located in Western Canada & USA (WCU), New Zealand (NZ), Scandinavia and Iceland (SI), Caucasus and Middle East (CME) and Central Europe (CE); with the basin name, region, and Global Runoff Data Centre (GRDC) index provided. For each basin, the glaciation extent around year 2000, the number of years with estimated trough water and the maximum difference (Δ_{max}) are indicated. Only three scenarios are presented to enhance comparison.

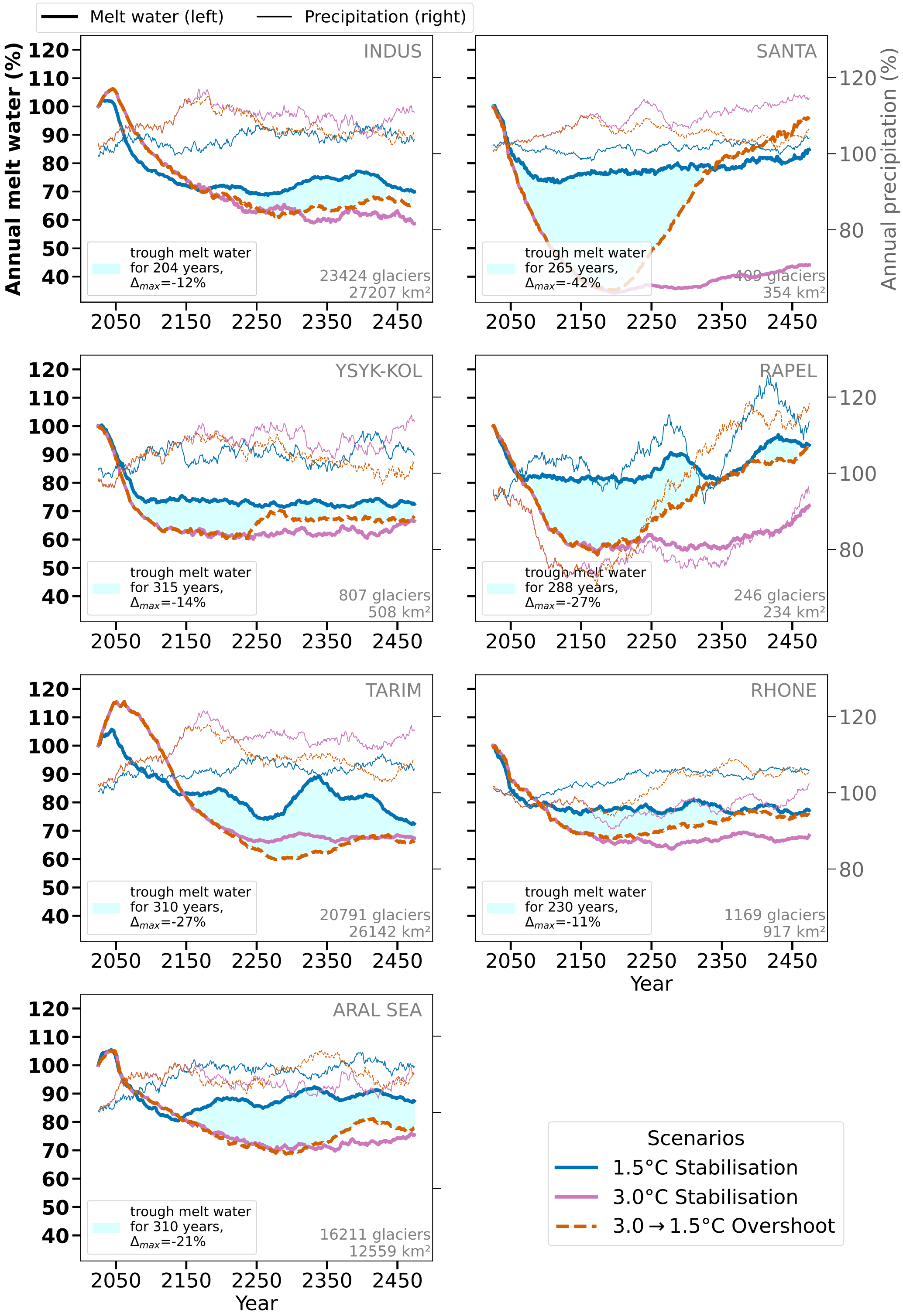

Supplementary Figure 6. Meltwater components of the annual glacier runoff projections (relative to 2000–2050) and respective near-glacier precipitation (relative to 2000–2019, both 51-year averaged) for the seven relatively arid and strongly glaciated basins. Similar to Fig. 4b but showing only the ice and snow melt on and off the glacierised area, thus discarding the liquid precipitation runoff components. The "trough melt water" was defined equivalent to the "trough water" with only the meltwater components. We give the number of glaciers together with the glaciated area, and the number of years with estimated trough water with the maximum difference (Δ_{max}). Only three scenarios are presented to enhance comparison. The map shows the outlines of the seven selected basins. All basins have an aridity index <2 (according to ref.[11]) and are initially above 0.9% glaciated.

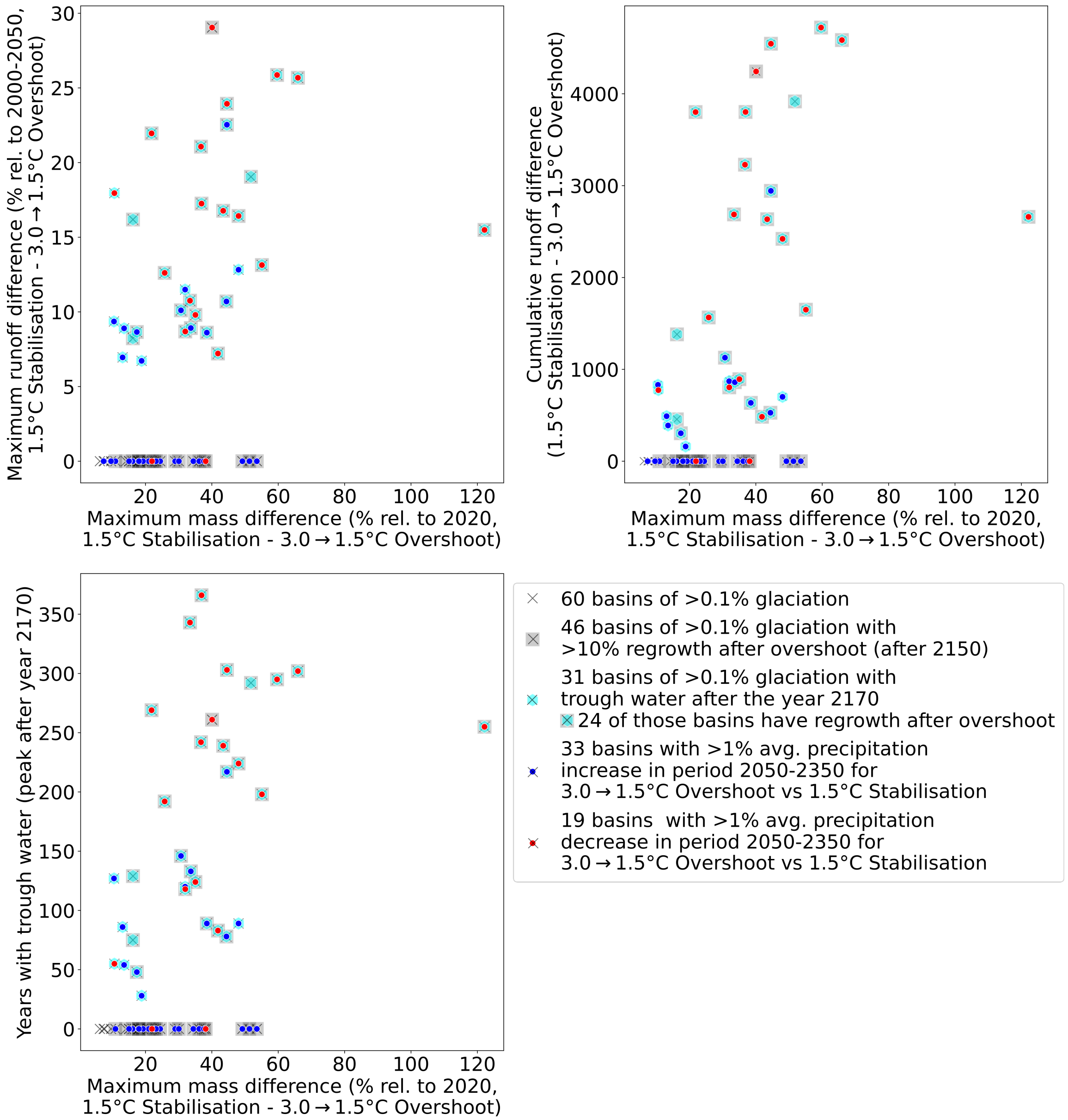

Supplementary Figure 7. **Basin statistics of annual glacier runoff differences (51-year averaged) between the 1.5°C Stabilisation and the 3.0→1.5°C Overshoot scenario for 60 glaciated basins**. The individual projections of these 60 basins are shown in Figs. 3-5. Basins where glacier mass regrows by at least 10% after year 2150 are highlighted together with those with a trough water that peaks after year 2170. We also show for each basin whether average precipitation increases or decreases in the 2050-2350 period under the overshoot scenario.

Annually averaged glacier runoff changes (Supplementary Figs. 3-5) are often not a very good characteristic of hydrological impacts of glacier changes[11], but still give an overview of the long-term patterns. 46 glaciated basins regrow by more than 10% after the temperature overshoot (after 2150), and 24 of those basins also create trough water after 2170 (Supplementary Fig. 7). For the 19 glaciated basins with regional precipitation decrease during a regional temperature overshoot (e.g. Rapel basin, GRDC index of 3423, in Southern Andes), the trough water is larger than on those 33 basins with an apparent

regional precipitation increase (e.g. Salween basin, 2319, in High Mountain Asia). For basins where precipitation increases with warming, total annual glacier runoff can increase and stabilise at higher levels in the longer term under warmer scenarios (e.g. Brahmaputra basin, 2302, in High Mountain Asia; Santa basin, 3425, in the Northern Andes; and Taku basin, 4431, in Western Canada & USA) due to increased amounts of liquid precipitation (Supplementary Figs. 3-5). For basins where precipitation decreases with warming (e.g. Cisnes basin, 3408, or Rapel basin, 3423, both in Southern Andes; and Rhone basin, 6243, in Central Europe), decreasing annual runoff and trough water may be intensified after the 21st century (Supplementary Figs. 3-5, 7).

When only considering the annual meltwater components of the glacier runoff (snow and ice melt on and off the glacier area), the direct effects of increased liquid precipitation from the scenario-dependent precipitation changes get removed (Supplementary Fig. 6). For all seven selected basins, the trough meltwater is more intense than the trough water from the annual glacier runoff (Supplementary Figs. 3-6). The effect of temperature-dependent precipitation changes on annual glacier runoff was similarly found in the idealised experiments (Supplementary Fig. 2).

Although we show here the glacier runoff response to a global temperature overshoot for every basin, we want to emphasise again that these projections only stem from a single glacier model and a single Earth System Model. Additional model simulations are necessary for more robust quantitative estimates.

Additional analysis on local or regional climate of W5E5 or GFDL-ESM2M

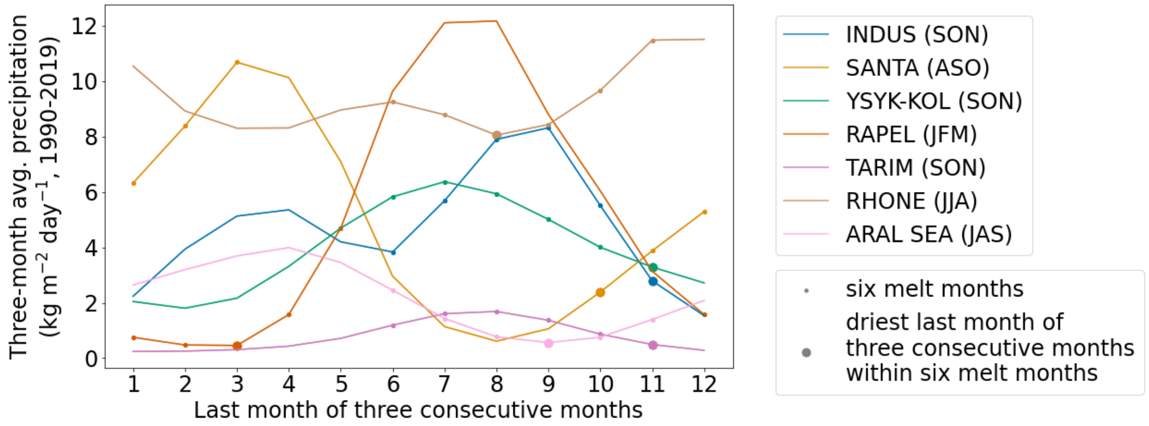

Supplementary Figure 8. Three-month averaged precipitation cycle and melt season of the seven relatively arid and strongly glaciated basins (1990–2019). The precipitation estimates were taken from the reanalysis climate dataset W5E5. The driest three-month period (given as the last month of three consecutive months) within the melt season (six months with the largest melt) is highlighted (see methods). The initial letters of the driest melt period months are also given in the legend.

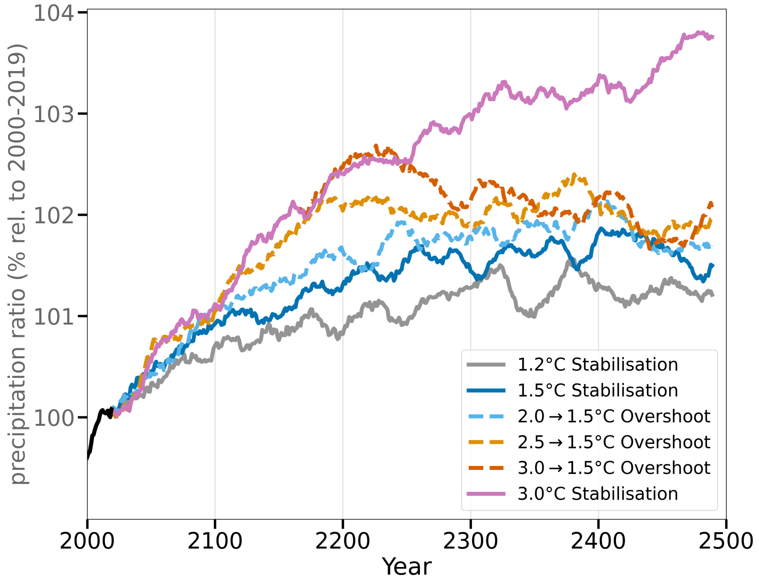

Supplementary Figure 9. **Global projected precipitation changes (21-year centred rolling mean) for the used stabilisation and overshoot scenarios of the GFDL-ESM2M.** Past changes from 2000 to 2019 are shown in black. The global temperature changes are in Fig. 2a.

Analysis of the bias correction period influence on glacier mass projections

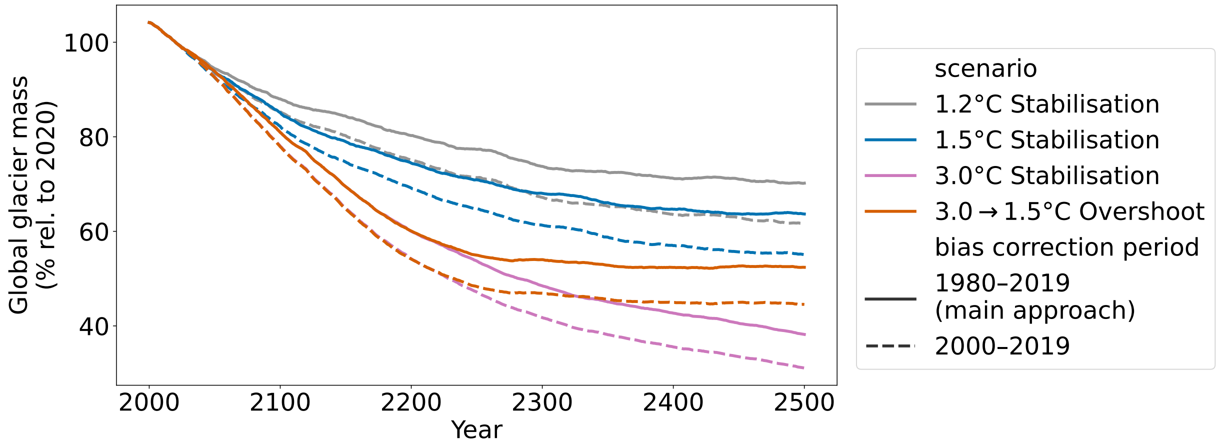

Supplementary Figure 10. **Global glacier mass remaining for two different bias correction periods and four stabilisation and overshoot scenarios**. The default bias-correction approach in our study is to use the longest available period (1980–2019). Other glacier projections, however, use the mass-balance calibration period, i.e. 2000–2019, as the bias correction period. For the comparison, we only used those glaciers that did not fail in any of the bias correction period approaches.

References

[1] Schuster, L., Chizzola, R., Huss, M., Maussion, F., Rounce, D. R., & Tober, B. S. *lilianschuster/glacier-model-projections-until2300: Glacier projection figures for the State of the Cryosphere 2023 and 2024 report [Code and Dataset] (v2024.0)*. Zenodo https://doi.org/10.5281/zenodo.10055416 (2024).

[2] Maussion, F., Butenko, A., Champollion, N., Dusch, M., Eis, J., Fourteau, K., Gregor, P., Jarosch, A. H., Landmann, J., Oesterle, F., Recinos, B., Rothenpieler, T., Vlug, A., Wild, C. T., & Marzeion, B. *The Open Global Glacier Model (OGGM) v1.1*. Geoscientific Model Development, 12(3), 909–931 (2019).

[3] Rounce, D. R., Hock, R., Maussion, F., Hugonnet, R., Kochtitzky, W., Huss, M., Berthier, E., Brinkerhoff, D., Compagno, L., Copland, L., Farinotti, D., Menounos, B., & McNabb, R. W. *Global glacier change in the 21st century: Every increase in temperature matters*. Science, 379(6627), 78–83 (2023).

[4] Zekollari, H., Huss, M., Schuster, L., Maussion, F., Rounce, D. R., Aguayo, R., Champollion, N., Compagno, L., Hugonnet, R., Marzeion, B., Mojtabavi, S., & Farinotti, D. *Twenty-first century global glacier evolution under CMIP6 scenarios and the role of glacier-specific observations*. The Cryosphere, 18, 50455066 (2024).

[5] Huss, M., & Hock, R. *A new model for global glacier change and sea-level rise*. Frontiers in Earth Science, 3, 54 (2015).

[6] Huss, M., & Hock, R. *Global-scale hydrological response to future glacier mass loss*. Nature Climate Change, 8(2), 135–140 (2018).

[7] Lacroix, F., Burger, F. A., Silvy, Y., Schleussner, C.-F., & Frölicher, T. L. *Persistently Elevated High-Latitude Ocean Temperatures and Global Sea Level Following Temporary Temperature Overshoots*. Earth's Future, 12(10), e2024EF004862 (2024).

[8] Schleussner, C.-F., Levermann, A., & Meinshausen, M. *Probabilistic projections of the Atlantic overturning*. Climatic Change, 127(3–4), 579–586 (2014).

[9] Herger, N., Sanderson, B. M., & Knutti, R. *Improved pattern scaling approaches for the use in climate impact studies*. Geophysical Research Letters, 42(9), 3486–3494 (2015).

[10] Gilbert, A., Flowers, G. E., Miller, G. H., Rabus, B. T., Van Wychen, W., Gardner, A. S., & Copland, L. *Sensitivity of Barnes Ice Cap, Baffin Island, Canada, to climate state and internal dynamics*. Journal of Geophysical Research: Earth Surface, 121(8), 1516–1539, (2016).

[11] Ultee, L., Coats, S., & Mackay, J. *Glacial runoff buffers droughts through the 21st century*. Earth System Dynamics, 13(2), 935–959 (2022).
